# Evidence for gene duplication in the voltage-gated sodium channel gene of *Aedes aegypti*

**DOI:** 10.1093/emph/eot012

**Published:** 2013-06-19

**Authors:** Ademir Jesus Martins, Luiz Paulo Brito, Jutta Gerlinde Birggitt Linss, Gustavo Bueno da Silva Rivas, Ricardo Machado, Rafaela Vieira Bruno, José Bento Pereira Lima, Denise Valle, Alexandre Afranio Peixoto

**Affiliations:** ^1^Laboratório de Fisiologia e Controle de Artrópodes Vetores, Instituto Oswaldo Cruz—FIOCRUZ and Laboratório de Entomologia, Instituto de Biologia do Exército, Rio de Janeiro, RJ, 21040-360, Brazil, ^2^Instituto Nacional de Ciência e Tecnologia em Entomologia Molecular, Brazil, ^3^Laboratório de Biologia Molecular de Insetos, Instituto Oswaldo Cruz—FIOCRUZ, Rio de Janeiro, RJ, 21040-360, Brazil and ^4^Laboratório de Biologia Molecular de Flavivirus, Instituto Oswaldo Cruz—FIOCRUZ, Rio de Janeiro, RJ, 21040-360, Brazil

**Keywords:** gene duplication, *kdr* mutation, sodium channel, pyrethroid resistance, *Aedes aegypti*

## Abstract

**Background and objectives:** Mutations in the voltage-gated sodium channel gene (*Na_V_*), known as *kdr* mutations, are associated with pyrethroid and DDT insecticide resistance in a number of species. In the mosquito dengue vector *Aedes aegypti*, besides *kdr*, other polymorphisms allowed grouping *AaNa_V_* sequences as type ‘A’ or ‘B’. Here, we point a series of evidences that these polymorphisms are actually involved in a gene duplication event.

**Methodology:** Four series of methods were employed: (i) genotypying, with allele-specific PCR (AS-PCR), of two *AaNa_V_* sites that can harbor kdr mutations (Ile1011Met and Val1016Ile), (ii) cloning and sequencing of part of the *AaNa_V_* gene, (iii) crosses with specific lineages and analysis of the offspring genotypes and (iv) copy number variation assays, with TaqMan quantitative real-time PCR.

**Results:** kdr mutations in 1011 and 1016 sites were present only in type ‘A’ sequences, but never in the same haplotype. In addition, although the 1011Met-mutant allele is widely disseminated, no homozygous (1011Met/Met) was detected. Sequencing revealed three distinct haplotypes in some individuals, raising the hypothesis of gene duplication, which was supported by the genotype frequencies in the offspring of specific crosses. Furthermore, it was estimated that a laboratory strain selected for insecticide resistance had 5-fold more copies of the sodium channel gene compared with a susceptible reference strain.

**Conclusions and implications:** The *AaNa_V_* duplication here found might be a recent adaptive response to the intense use of insecticides, maintaining together wild-type and mutant alleles in the same organism, conferring resistance and reducing some of its deleterious effects.

## Abstract (precis)

Herein, we show the first evidence of a duplication of the NaV gene of the mosquito Aedes aegypti, that might be involved in insecticide resistance. The duplicated haplotype is composed of one sequence with and another without a specific mutation, present in natural populations as a polymorphic trait.

## BACKGROUND AND OBJECTIVES

The use of DDT as public health insecticide was one of the factors responsible for the yellow fever mosquito eradication in many Latin American countries in the 1950s [1]. Since the reintroduction of *Aedes aegypti* to South America, organophosphates and, subsequently, pyrethroid insecticides have been extensively used in governmental campaigns as well as in residential or private services. Pyrethroids have similar effects as DDT but with a lower residual effect in the environment, and they represent nowadays the main class of insecticide against arthropods, not only those of medical and veterinary importance but also in relation to agriculture and livestock [2]. In Brazil, despite the recent introduction of pyrethroids in campaigns for dengue control throughout the whole country, resistance to these compounds has already been detected in many *Ae. aegypti* populations [3, 4].

Pyrethroids and DDT have a rapid effect on the insect central nervous system, leading to repetitive and involuntary muscular contractions, followed by paralysis and death, commonly reported as knockdown effect [5, 6]. Accordingly, resistance to this is referred to as knockdown resistance (*kdr*), the principal cause being a mutation in the pyrethroid/DDT target site, the voltage-gated sodium channel (Na_V_). The Na_V_ is an axonic transmembrane protein composed of four homologous domains (I–IV), each one with six hydrophobic segments (S1–S6) [7]. To date, most of the *kdr* mutations described lie in the Na_V_ IIS6 region, and the Leu/Phe substitution in the 1014 site (numbered according to the *Musca domestica* amino acid primary sequence) is by far the most common among all studied insects. Relatively recent analyses of *kdr* mutations in a series of arthropod species contributed to the knowledge concerning evolution and dynamics of pyrethroid resistance in natural populations. This effort is essential to formulate strategies able to prolong the effectiveness of pyrethroids in the field and to develop new compounds targeting the sodium channel [8, 9]. Some extensive reviews of *kdr* mutations are available [2, 10, 11].

Several mutations have been identified in the *Ae. aegypti Na_V_* gene (*AaNa_V_*) comprising the IIS5–S6 region: Gly923Val, Leu982Trp, Ile1011Met, Ile1011Val, Val1016Ile and Val1016Gly [12–16]. The Ile1011Met substitution was associated with low sensitivity to pyrethroids evidenced by electrophysiological assays [12] and was the most frequent in a resistant Brazilian natural *Ae. aegypti* population [14]. However, substitutions in another position, 1016 (Val/Ile in South and Central America and Val/Gly in Thailand), are presently attributed with a more important role in pyrethroid resistance, the 1016 substitutions appearing as a recessive trait [13, 16–18]. Outside domain II, a Phe1534Cys substitution in the IIIS6 region was also related to pyrethroid resistance [19]. Besides amino acid changes, nucleotide and insertion/deletion polymorphisms have been detected in intron 20 in the *AaNa_V_* IIS6 genomic region that enable grouping the sequences in two categories, type ‘A’ or type ‘B’. The Ile1011Met and Val1016Ile mutations are found only in type ‘A’ sequences [14].

Herein, we further investigated the nature of this polymorphism. Sequencing of the *AaNa_V_* IIS6 genomic region and alelle specific-PCR (AS-PCR) typing of the 1011 and 1016 sites revealed, in several cases, three haplotypes in the same mosquito. Besides, in no case were homozygous specimens for the 1011Met mutation in natural populations detected. Crosses between laboratory-selected genotypes and copy number variation assays strongly suggested the occurrence of duplication events in the sodium channel gene, at least for the studied genomic region.

## MATERIALS AND METHODS

### Mosquitoes

Rockefeller strain, continuously reared in the laboratory as a standard for insecticide susceptibility and life-history trait parameters, was used as reference for wild-type alleles for the voltage-gated sodium channel gene. The EE lineage was originated from laboratory selection pressure for nine consecutive generations with the pyrethroid deltamethrin using a sample of a natural population from Natal (a locality from the Northeast of Brazil) that did not harbor the mutation in the 1016 site [20]. Rearing and maintenance of the colonies were conducted according to standard laboratory conditions [21]. Field populations were obtained by sampling as described elsewhere [13].

### Molecular assays

Genotyping by allele-specific PCR (AS-PCR) for the *AaNa_V_* 1011 site and sequencing of the IIS6 genomic region were performed with the DNA from the same specimens genotyped for the 1016 alleles, described in a previous report [13]. PCR discriminating type ‘A’ or ‘B’ sequences (see [14]) was carried out in 12.5 µl reactions containing 1 µM of each primer ‘forward’ (5′-AGGCTGACTGAAAGTAAATTGG-3′) and ‘reverse’ (5′-CAAAAGCAAGGCTAAGAAAAGG-3′), 6.25 µl of GoTaq Green Master Mix 2X (Promega) and 0.5 µl of genomic DNA, submitted for 30 denaturation, annealing and extension cycles under, respectively, 94°C/30", 60°C/1' and 72°C/45". The amplified region includes the intron 20, polymorphic in size, in the *AaNa_V_* IIS6 region. For the 1011 site genotyping, PCR with 0.24 µM of common and 0.12 µM of each of the two specific primers [17] was performed as above, with 30 cycles of denaturation, annealing and extension under, respectively, 94°C/30", 57°C/1' and 72°C/45” conditions. The PCR products were analyzed in 10% polyacrylamide gel electrophoresis stained in 1 µg/ml ethidium bromide solution. The *AaNa_V_* IIS6 region was amplified, cloned and sequenced as previously reported [14] in individual specimens from Uberaba, Cuiabá, Aparecida de Goiânia, Maceió and Fortaleza. Sequences of at least eight clones of each insect were analyzed.

The numbers of copies of the *AaNa_V_* IIS6 genomic region were compared among the Rockefeller strain, the EE lineage and their F1 offspring (Hyb). DNA was extracted from pools of 10 L3 larvae (∼20 mg) with the kit Insect DNA Extraction (Zymo Research) according to the manufacturer’s instructions, brought to 5 ng/μl in H_2_O and aliquoted. Real-time PCR reactions were carried out based on instructions of customized TaqMan Copy Number Assay (Applied Biosystems) in 15 μl, containing 7.5 μl of 2× TaqMan Genotyping Master Mix (Applied Biosystems), 0.75 μl of 20× mix composed of primers and probes for both target and reference genes, 20 ng of DNA and H_2_O. The chosen single copy reference was the ribosomal gene *RP49* (GenBank accession number AY539746), with primers AaRP49_F: 5′-ACATCGGTTACGGATCGAACAAG-3′, AaRP49_R: 5′-TGTGGACCAGGAACTTCTTGAAG-3′ and probe AaRP49_M: 5′-VIC-CACCCGCCATATGCT-MGB-NFQ-3′. The target was determined based on the *AaNa_V_* IIS6 region (GenBank accession number FJ479613) with primers AaNa_V_ex20_F: 5′-ACCGACTTCATGCACTCATTCAT-3′, AaNa_V_ex20_R: 5′-ACAAGCATACAATCCCACATGGA-3′ and probe AaNa_V_ex20_M: 5′-FAM-CCACTCGCCGCATAAT-MGB-NFQ-3′. Three assays were performed with DNA from three distinct pools of each lineage, in triplicate/assay. Reactions were conducted in an ABI StepOne Thermocycler (Applied Biosystems), following standard cycling conditions for TaqMan Genotyping assays. The C_T_s for the target (*AaNa_V_*) and reference (*RP49*) genes were determined based on automatic threshold indicated by the StepOne Software v2.0. Given the C_T_ of each sample, their ΔC_T_s were established, intended to normalize the amount of amplified products from *AaNa_V_* by *RP49*, and then the average of the replicates from each pool ΔC_T_ (*μ*[ΔC_T_]) was calculated. The ΔΔC_T_ of the test lineages (EE and Hyb) were obtained by the difference between their *μ*[ΔC_T_] and that of Rockefeller. Finally, the average of ΔΔC_T_s from the three assays (*μ*[ΔΔC_T_]) was calculated in order to estimate the number of *AaNa_V_* copies, normalized by *RP49*, related to Rockefeller. The diploid number of the target sequence of the tested sample was determined by the formula: cn_c_2^ΔΔCT^, where cn_c_ is the copy number of the target sequence in the reference sample and ΔΔC_T_ is the difference between the ΔC_T_ for the tested sample and the reference sample.

### Crossing experiments

Crosses were performed between mosquitoes from Rockefeller and EE strains, respectively, homozygous (Ile/Ile) and apparently ‘heterozygous’ (Ile/Met) for the 1011 site. Each couple of one male and one virgin female was maintained for at least 3 days in conical 50 ml tubes covered with a mesh tulle under a cotton wool soaked in sugar solution. Females were then blood-fed on anesthetized mice, 24 h after sugar removal. Individual females were induced to lay eggs in small Petri dishes lined with wet filter paper [22]. Resulting F1 larvae were reared until adults for genotyping by AS-PCR or for subsequent crossings to obtain F2, performed as above.

### Ethics statement

#### Mosquito blood feeding

*Aedes aegypti* females were fed on anesthetized mice (ketamine:xylazine 80–120:10–16 mg/kg), according to institutional procedures, oriented by the national guideline ‘the Brazilian legal framework on the scientific use of animals’ [23]. This study was reviewed and approved by the Fiocruz Ethics Committee on Animal Use (CEUA/FIOCRUZ), license number: L-011/09.

#### Entomological survey

All field egg collections were conducted by agents from each respective State Health Secretariat, following procedures designed by the National Program of Dengue Control/Brazilian Ministry of Health. All ovitraps were installed and collected in the houses with residents’ permission.

## RESULTS

### Typing of 1011 and 1016 sodium channel sites in *Ae. aegypti* natural populations by AS-PCR

The allele frequencies of the *AaNa_V_* 1011 site were evaluated in the same mosquitoes which had the 1016 site analyzed previously, belonging to samples from 15 Brazilian localities [13]. The 1011Met-mutant allele was found in all localities, except in Boa Vista. In seven localities, specimens were divided into pyrethroid susceptible (S) or resistant (R) [13]. Table 1 shows allele frequencies considering both 1011 and 1016 sites together, combined in six molecular phenotypes, derived from three potential haplotypes (1011Ile + 1016Val, 1011Ile + 1016Ile and 1011Met + 1016Val). We assumed that the recombinant haplotype containing both mutant alleles (1011Met + 1016Ile) was not expected, because these sites are very close in the genome and both mutations are likely to be very recent. We observed that the 1011Ile/Ile + 1016Ile/Ile combination, i.e. homozygous for the wild-type and for the mutant allele, respectively, in the 1011 and 1016 sites, was far more frequent among resistant than susceptible insects. This suggests that the 1016 site is probably more important for pyrethroid resistance than the 1011 site.

**Table 1.** Phenotypic frequency, considering *AaNa_V_* 1011 and 1016 sites, of *Ae. aegypti* natural populations from Brazil

Locality	Status	*n*	Frequency of genotypes: observed (and expected assuming Hardy–Weinberg equilibrium)						
			1011Ile/Ile			1011Ile/Met		1011Met/Met	HWE
			1016Val/Val	1016Val/Ile	1016Ile/Ile	1016Val/Val	1016Val/Ile	1016Val/Val	χ^2^, df, *P*
Aparecida de Goiânia	R	18	0.056 (0.094)	0 (0.204)	0.222 (0.111)	0.500 (0.221)	0.222 (0.240)	0 (0.130)	14.7, 5, 0.0119
	S	19	0.105 (0.305)	0.053 (0.029)	0 (0.001)	0.842 (0.465)	0 (0.022)	0 (0.177)	2.9, 5, 0.7204
Campo Grande	R	22	0.045 (0.052)	0.273 (0.299)	0.455 (0.435)	0.091 (0.052)	0.136 (0.150)	0 (0.013)	1.1, 5, 0.9571
	S	17	0.118 (0.221)	0.118 (0.138)	0 (0.022)	0.588 (0.360)	0.176 (0.112)	0 (0.146)	6.8, 5, 0.2347
Cuiabá	R	13	0.231 (0.148)	0 (0.325)	0.385 (0.179)	0.308 (0.148)	0.077 (0.163)	0 (0.037)	11.2, 5, 0.0473
	S	14	0.571 (0.617)	0.143 (0.112)	0 (0.005)	0.286 (0.020)	0 (0.020)	0 (0.037)	1.0, 5, 0.9589
Dourados	R	16	0 (0.035)	0.063 (0.223)	0.500 (0.353)	0.313 (0.260)	0.125 (0.048)	0 (0.037)	15.6, 5, 0.0080
	S	20	0.250 (0.303)	0.250 (0.275)	0.100 (0.063)	0.350 (0.220)	0.050 (0.100)	0 (0.040)	3.5, 5, 0.6213
Fortaleza	R	16	0.250 (0.391)	–	–	0.750 (0.469)	–	0 (0.141)	5.8, 2, 0.0561
	S	16	0.313 (0.431)	–	–	0.688 (0.451)	–	0 (0.118)	4.4, 2, 0.1114
Maceió	R	15	0.467 (0.538)	–	–	0.533 (0.391)	–	0 (0.071)	2.0, 2, 0.3709
	S	15	0.333 (0.444)	–	–	0.667 (0.444)	–	0 (0.111)	3.8, 2, 0.1534
Uberaba	R	23	0.043 (0.030)	0.087 (0.204)	0.391 (0.345)	0.174 (0.083)	0.304 (0.281)	0 (0.057)	5.5, 5, 0.3619
	S	20	0.300 (0.276)	0.050 (0.184)	0.050 (0.031)	0.400 (0.315)	0.200 (0.240)	0 (0.090)	6.2, 5, 0.2860
Boa Vista	*	20	0.950 (0.930)	0 (0.095)	0.050 (0.003)	–	–	–	1.9, 2, 0.3772
Cachoeiro do Itapemirim	*	20	0.200 (0.090)	0 (0.255)	0.250 (0.181)	0.200 (0.165)	0.350 (0.234)	0 (0.076)	11.1, 5, 0.0487
Colatina	*	16	0 (0.191)	0.250 (0.191)	0.063 (0.048)	0.625 (0.301)	0.063 (0.150)	0 (0.118)	11.7, 5, 0.0388
Foz do Iguaçu	*	19	0 (0.003)	0.053 (0.078)	0.526 (0.543)	0.053 (0.022)	0.368 (0.310)	0 (0.044)	2.1, 5, 0.8408
Ijuí	*	20	0.900 (0.903)	–	–	0.100 (0.095)	–	0 (0.003)	0.06, 5, 0.9727
Macapá	*	20	0.300 (0.423)	–	–	0.700 (0.455)	–	0 (0.123)	5.8, 2, 0.0551
Santa Bárbara	*	16	0.938 (0.938)	–	–	0.063 (0.061)	–	0 (0.001)	0.02, 2, 0.9917
Santa Rosa	*	20	0.650 (0.681)	–	–	0.350 (0.289)	–	0 (0.031)	0.9, 2, 0.6377

Frequencies observed and expected (for Hardy–Weinberg equilibrium) of the molecular phenotypes derived by AS-PCR for the sites 1011 and 1016 in the same insects. In the header, the mutant alleles are underlined. Some populations are divided regarding their resistant (R) or susceptible (S) status to pyrethroid resistance. Populations whose individuals were not divided in R or S are marked with an asterisk (*) in status. The absence of the mutations 1011Ile/Met and 1016Val/Ile in a population is represented as endash (–). The last column gives the result of χ^2^ analyses for testing Hardy–Weinberg equilibrium (HWE). The 1016 genotyping data were already presented elsewhere [13].

Two other striking results can also be observed. First, we did not detect any specimen ‘homozygous’ for the 1011Met (1011Met/Met + 1016Val/Val) mutation. Second, there is a higher than expected frequency of the 1011Ile/Met + 1016Val/Val molecular phenotype in all samples, except the near monomorphic Boa Vista population (Table 1). Although the individual tests of the Hardy–Weinberg expectations for each sample were significant only in four cases, likely due to the small sample sizes, the lack of the 1011Met/Met + 1016Val/Val molecular phenotype and the excess of 1011Ile/Met + 1016Val/Val were observed in almost all populations. Two simple hypotheses were considered to explain this pattern. One possibility is that the 1011Met mutation is involved in a gene duplication, carrying both the mutant (1011Met + 1016Val) and the wild-type allele (1011Ile + 1016Val). In this case, the 1011Met/Met genotype would never be detected by the AS-PCR, because that duplication would generate a molecular phenotype mimicking a heterozygous 1011Ile/Met. Alternatively, one might argue that the 1011Met mutation is lethal when in homozygosis. However, this is not the case ([16], see ‘Discussion’ section herein), and it does not explain the increased frequency of 1011Ile/Met + 1016Val/Val, unless one also assumes this particular combination has a higher fitness. In order to better understand these data, we cloned and sequenced the IIS6 region from a number of mosquitoes.

### Sequencing of the IIS6 region of the *Ae. aegypti* sodium channel gene

**Figure 1.**
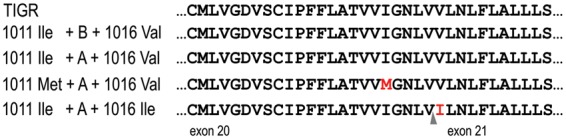
Diversity of a voltage-gated sodium channel gene region observed in *Ae. aegypti* Brazilian populations. Part of the region corresponding to the *AaNa_V_* exons 20 and 21, and the intron between them, are represented. A and B indicate the type of intron, as previously stated [14]. In red, the presumed amino acids for the sites 1011 and 1016. Genomic sequences representative for each haplotype were submitted to GenBank: 1011Ile + B + 1016Val (GenBank accession number: FJ479613), 1011Ile + A + 1016Val (FJ479611), 1011Met + A + 1016Val (FJ479612) and 1011Ile + A + 1016Ile (JX275501). TIGR = sequence from *Ae. aegypti* genome project (Vectorbase)

We obtained sequences of the *AaNa_V_* IIS6 region from a number of mosquitoes from five Brazilian populations (see ‘Materials and Methods’ section for details) and confirmed the polymorphism in this genomic region. Figure 1 shows the haplotypes and their respective submission numbers in GenBank. Sequences were classified as ‘A’ or ‘B’, according to two synonymous substitutions in exon 20 and differences in the intron (see [14] for details). The Ile1011Met substitution was seen in all studied populations, whereas Val1016Ile was not detected in the Northeastern localities (Maceió and Fortaleza). Both substitutions were present only in sequences type ‘A’, and among sequences from 40 individuals, no haplotype shared substitutions in both the 1011 and 1016 sites, indicating no recombinants between the two mutations. As mentioned above, this was expected considering that these sites are very close, and the mutations are likely to be very recent. Hence, only four haplotypes were observed (1011Ile + A + 1016Val, 1011Ile + A + 1016Ile, 1011Ile + B + 1016Val and 1011Met + A + 1016Val) out of six possibilities, considering the type of sequence (‘A’ or ‘B’) and the sites 1011 (Ile or Met) and 1016 (Val or Ile) (Table 2). Moreover, the 1011Met + A + 1016Val haplotype was only present in specimens which also harbored the 1011Ile + B + 1016Val haplotype, therefore, classified as ‘heterozygous’. Accordingly, typing of various natural populations had revealed the absence of ‘homozygous’ for the 1011Met mutation (Table 1). Curiously, some specimens presented three haplotypes, which were in all cases: 1011Met + A + 1016Val, 1011Ile + A + 1016Ile and 1011Ile + B + 1016Val (Table 2). It is important to mention that females had their abdomen removed prior to DNA extraction in order to avoid eventual amplification of DNA from spermatozoids stored in the spermatechae, and there was no evidence of contamination in PCR negative controls. The last column of Table 2 presents the expected ‘genotypes’ through sequence typing (A or B) and the 1011 and 1016 sites. Sequencing confirmed the results for all insects genotyped by AS-PCR (data not shown).

**Table 2.** Sequencing of the *AaNa_V_* IIS6 genomic region of specimens from *Ae. aegypti* Brazilian natural populations

Locality	Sample	Haplotype (1011 + intron + 1016)						Molecular phenotype (1011 + intron + 1016)
		Ile	**Met**	Ile	Ile	**Met**	Ile	
		+	+	+	+	+	+	
		A	A	A	B	B	B	
		+	+	+	+	+	+	
		Val	Val	**Ile**	Val	Val	**Ile**	
Uberaba	UBR-04	X			X			Ile/Ile + AB + Val/Val
	UBR-08		X		X			Ile/Met + AB + Val/Val
	UBR-10		X	X	X			Ile/Met + AB + Val/Ile
	UBR-S25	X		X				Ile/Ile + AA + Val/Ile
	UBR-S26	X		X				Ile/Ile + AA + Val/Ile
	UBR-R1			X				Ile/Ile + AA + Ile/Ile
	UBR-R3			X				Ile/Ile + AA + Ile/Ile
	UBR-R10				X			Ile/Ile + BB + Val/Val
	UBR-R11			X	X			Ile/Ile + AB + Val/Ile
	UBR-R13		X	X	X			Ile/Met + AB + Val/Ile
	UBR-R20			X				Ile/Ile + AA + Ile/Ile
	UBR-R22			X				Ile/Ile + AA + Ile/Ile
	UBR-R26			X				Ile/Ile + AA + Ile/Ile
Cuiabá	CUI-01			X	X			Ile/Ile + AB + Val/Ile
	CUI-02	X						Ile/Ile + AA + Val/Val
	CUI-03	X			X			Ile/Ile + AB + Val/Val
	CUI-04	X			X			Ile/Ile + AB + Val/Val
	CUI-07	X			X			Ile/Ile + AB + Val/Val
	CUI-08	X			X			Ile/Ile + AB + Val/Val
	CUI-12	X			X			Ile/Ile + AB + Val/Val
	CUI-R16	X			X			Ile/Ile + AB + Val/Val
	CUI-S15		X		X			Ile/Met + AB + Val/Val
Ap Goiânia	APG-01	X		X				Ile/Ile + AA + Val/Ile
	APG-02		X	X	X			Ile/Met + AB + Val/Ile
	APG-04		X		X			Ile/Met + AB + Val/Val
	APG-05		X		X			Ile/Met + AB + Val/Val
	APG-06		X		X			Ile/Met + AB + Val/Val
	APG-07		X	X	X			Ile/Met + AB + Val/Ile
	APG-08		X		X			Ile/Met + AB + Val/Val
	APG-09		X		X			Ile/Met + AB + Val/Val
	APG-10		X		X			Ile/Met + AB + Val/Val
	APG-11		X		X			Ile/Met + AB + Val/Val
	APG-12		X		X			Ile/Met + AB + Val/Val
Maceió	COM-02		X		X			Ile/Met + AB + Val/Val
	COM-07				X			Ile/Ile + BB + Val/Val
	COM-09				X			Ile/Ile + BB + Val/Val
Fortaleza	hrjg-21		X		X			Ile/Met + AB + Val/Val
	hrjg-22	X						Ile/Ile + AA + Val/Val
	hrjg-23				X			Ile/Ile + BB + Val/Val
	hrjg-28	X			X			Ile/Ile + AB + Val/Val

Identification of each sample corresponds to the sampling locality: UBR, Uberaba; CUI, Cuiabá; APG, Aparecida de Goiânia; COM, Maceió and hrjg, Henrique Jorge (a district of Fortaleza). ‘Haplotypes’ indicate the combination among site 1011 (Ile or Met) + type of intron (A or B) + site 1016 (Val or Ile). The haplotype observed for each insect is marked by an ‘X’. In the header, the mutations are indicated in bold letters. The last column shows the phenotypic classification, confirmed by AS-PCR.

**Figure 2.**
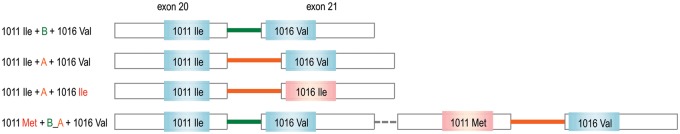
Schematic representation of *AaNa_V_* haplotypes. Blue boxes indicate exons 20 and 21 with the intron between them, the latter used to classify the haplotypes as A (orange) or B (green). Sites 1011 and 1016 are represented by the variant wild-type (blue box) or mutant (red box). According to our hypothesis, there is a duplication in some populations, comprised of haplotypes 1011Ile + B + 1016Val and 1011Met + A + 1016Val. Dashed line suggests linkage of the haplotypes, but which one is upstream was not determined

The presence of three alleles in one specimen suggests the gene duplication, at least in the genomic region analyzed. However, search in the *Ae. aegypti* genome project database (http://aaegypti.vectorbase.org/) did not indicate any evidence that the original Liverpool strain has more than one copy of any part, let alone the whole voltage-gated sodium channel gene. Based on the available sequences, this strain would be classified as homozygous for the 1011Ile + B + 1016Val allele, just like the Rockefeller strain used here. Hence, the putative duplication does not occur in all individuals, being therefore a polymorphic trait. In the samples analyzed, we detected mosquitoes ‘homozygous’ for the 1011Ile + B + 1016Val, 1011Ile + A + 1016Val and 1011Ile + A + 1016Ile haplotypes, all having the wild-type allele for the 1011 site. However, the ‘1011Met + A + 1016Val’ (mutant in the 1011 site) haplotype was never detected in ‘homozygosis’, but always in association with ‘1011Ile + B + 1016Val’, suggesting that the duplication involves these two variants (Table 2). Figure 2 presents a schematic representation of *AaNa_V_* haplotypes proposed for the populations analyzed based on our duplication hypothesis. The offspring of crosses between some combinations of parental genotypes was further analyzed in order to test this hypothesis.

### Crossing experiments

**Figure 3.**
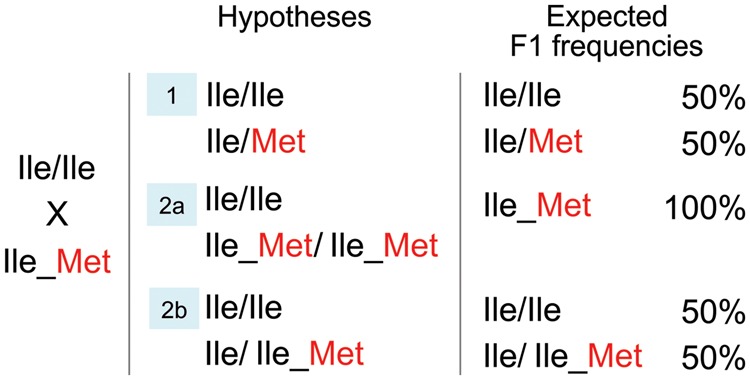
Three hypotheses with the expected genotypes and molecular phenotypes in the *AaNa_V_* 1011 site for the parental and their respective expected frequency in the F1 offspring. The 1011Met mutation is shown in red. See text for further details

In order to test the duplication hypothesis, we performed crosses between specimens with known molecular phenotypes (based on AS-PCR) and determined the frequency of the variants in the *AaNa_V_* 1011 site in their offspring. Initially, we evaluated the F1 of seven couples, each composed of a homozygous wild-type (1011Ile/Ile) and a putative heterozygous or duplicated (1011Ile/Met) progenitor, belonging, respectively, to the Rockefeller and the EE lineages. The latter originated from a laboratory population selection for pyrethroid resistance using a sample from a natural population that did not harbor the mutation Val1016Ile [20]. The results are shown in Table 3, with expected values and the Fisher tests for the three different hypotheses in Fig. 3, assuming either a duplication or no duplication. If the 1011Ile/Met parent did not harbor the duplicated haplotype, the offspring would present the Ile/Ile and Ile/Met genotypes in equal frequencies (Hypothesis 1). Assuming the occurrence of a duplication, one would expect the offspring genotyped as either 100% Ile/Met or alternatively Ile/Ile and Ile/Met in equal frequencies, respectively, if the parent was homozygous (Hypothesis 2a) or heterozygous (Hypothesis 2b) for the duplicated haplotype (Fig. 3).

**Table 3.** Testing the gene duplication hypothesis: molecular phenotype frequencies for the *AaNa*_v_ 1011 site in F1 offspring from crossings between *Ae. aegypti* Ile/Ile X Ile/Met

Crossings	F1 observed (*n*)		Hypotheses^a^								
			Without duplication			With duplication					
			Hypothesis 1			Hypothesis 2a			Hypothesis 2b		
	Ile/Ile	Ile/Met	Ile/Ile	Ile/Met	*P*	Ile/Ile	Ile/Met	*P*	Ile/Ile	Ile/Met	*P*
#1 (♀ Ile/Met x ♂ Ile/Ile)	0	20	10	10	***	0	20	NS	10	10	***
#2 (♀ Ile/Met x ♂ Ile/Ile)	0	20	10	10	***	0	20	NS	10	10	***
#3 (♀ Ile/Met x ♂ Ile/Ile)	8	12	10	10	NS	0	20	**	10	10	NS
#4 (♀Ile/Ile x ♂ Ile/Met)	9	9	9	9	NS	0	18	***	9	9	NS
#5 (♀ Ile/Ile x ♂ Ile/Met)	0	30	15	15	***	0	30	NS	15	15	***
#6 (♀ Ile/Met x ♂ Ile/Ile)	0	30	15	15	***	0	30	NS	15	15	NS
#7 (♀ Ile/Met x ♂ Ile/Ile)	0	22	11	11	***	0	22	***	11	11	NS

Molecular phenotype frequencies were determined by AS-PCR for the *AaNa_V_* 1011 site (see ‘Materials and Methods’ section). ^a^Expected numbers of F1 individuals of each molecular phenotype based on the three hypotheses of parental haplotype constitution (Fig. 3). Significance of the deviations of the tested hypotheses obtained through Fisher’s exact test: NS = non-significant, **P < 0.01, ***P < 0.001.

Two out of seven crosses (#3 and #4) had the 1011Ile/Ile genotype in around half of their offspring, which was thus not informative. In these two cases, this could be explained if the progenitor harboring the 1011Met mutation was heterozygous for the duplication (1011Ile/Ile_Met) as well as if it was heterozygous for non-duplicated haplotypes. However, as all the offspring from the other five crosses were 1011Ile/Met, the progenitor who harbored the mutation was necessarily homozygous for the duplication (Ile_Met/Ile_Met) (Fig. 3). In addition, the F2 offspring from crosses #1 (#1.1) and #2 (#2.1) revealed segregation in the approximated proportion of 3Ile/Met:1Ile/Ile (Table 4), corroborating the duplication hypothesis.

**Table 4.** Testing the gene duplication hypothesis: molecular phenotype frequencies for the *AaNa*_v_ 1011 site in F2 offspring from crosses #1 and #2 (Table 3)

Crossings (F1)	F2 (*n*)				
	Observed		Expected		
	Ile/Ile	Ile/Met	Ile/Ile	Ile/Met	*P*
#1.1 (♀ Ile/Met x ♂ Ile/Met)	5	25	8	22	NS
#2.1 (♀ Ile/Met x ♂ Ile/Met)	7	23	8	22	NS

Observed and expected numbers for each molecular phenotype in the F2 of crosses #1 and #2 (Table 3) assuming parents carry the following haplotypes Ile/Ile_Met × Ile/Ile_Met, in agreement with the duplication hypothesis (Fig. 3). The expected frequencies are 0.25 Ile/Ile and 0.75 Ile/Met (0.50Ile/Ile_Met + 0.25Ile_Met/Ile_Met). Deviations from the proposed hypotheses are non-significant (Fisher’s exact test; *P* > 0.05).

### Copy number assay

We analyzed the *AaNa_V_* copy number variation through molecular assays using DNA from pools of larvae from the Rockefeller reference strain, homozygous for the wild-type alleles, and a strain (EE) selected in the laboratory for pyrethroid resistance [20] and harboring the putative duplication in the *AaNa_V_*, as suggested by the assays described above. In this sense, we assessed the relative amount of DNA molecules containing the genomic region spanning the *AaNa_V_* 1011 site normalized by a reference gene (*RP49*). Assuming that the Rockefeller strain has only two copies of *AaNa_V_* as expected for a diploid with a single copy gene, the EE lineage selected for resistance and ‘homozygous’ for the duplication, revealed to have in fact 10 copies (Table 5 and Supplementary Table S1). Accordingly, the F1 resulting from Rockefeller and EE had six copies. The results therefore indicate further duplication events and amplification in this locus.

**Table 5.** Copy number variation assay for *AaNa_V_*

Assay	Rock			EE				Hib			
	*μ*[ΔC_T_]	(SD)	ΔΔCт	*μ*[ΔC_T_]	(SD)	ΔΔCт		*μ*[ΔC_T_]	(SD)	ΔΔCт	
1	−0.4	(0.09)	0	−2.7	(0.03)	−2.3		−2	(0.07)	−1.6	
2	0	(0.11)	0	−2.4	(0.04)	−2.4		−1.7	(0.05)	−1.6	
3	-0.7	(0.07)	0	−3	(0.04)	−2.4		-2.4	(0.06)	−1.7	
*μ*[ΔΔC_T_] (SD)			0			−2.3	(0.03)			−1.6	(0.07)
Cn			2			10				6	

Average and standard deviation ΔC_T_ (target − reference) followed by the ΔΔCт (lineage test − Rock) values from each lineage in each assay. Bottom: mean and standard deviation of ΔΔC_T_ from the three assays and the resulting number of copies (cn) of *AaNa_V_* relative to *rp49*.

## DISCUSSION

DDT and pyrethroids target the voltage-gated sodium channel (Na_V_) of insects, a key component of axon membranes exhibiting a fundamental physiological function in neural current propagation, with a complex but highly conserved structure among animals [24]. Vertebrate genomes present 6–10 Na_V_-coding genes, whereas invertebrate classes, such as Cnidaria and Annelida, have only 2–4 Na_V_ genes [25]. In insects, there is only one *Na_V_*, also commonly referred to as ‘paralytic’ *(para)*, due to its relationship with the phenotype of reversible paralysis under high temperatures in *Drosophila melanogaster-*mutant lineages [26, 27]. An important source of Na_V_ protein variability in different tissues relies on alternative splicing and RNA editing [28]. However, to date no association between pyrethroid resistance and variation derived from post-transcriptional modifications in the *Ae. aegypti Na_V_* gene has been uncovered [18]. Another possible source of molecular diversity might be polymorphism generated by recent gene duplications. Putative additional *Na_V_* in insects (the orthologous channels *DSC1* in *D. melanogaster* and *BSC1* in *Blattella germanica*) were later grouped close to calcium channels, both functionally and evolutionarily [29, 30]. Recently, two Na_V_ distantly related proteins were characterized in the *Periplaneta americana* cockroach, coded by the *PaNa_V_* and *PaFPC* para-like genes, a finding that suggested a possible early duplication event and subsequent loss of the *Na_V_* gene in some lineages [31].

The role of gene duplication and/or amplification in insecticide resistance has been described in at least 10 arthropod species, including mosquitoes [32]. The most classic case involves overexpression of *Culex* Esterase genes, leading to organophosphate resistance. This is the consequence of duplication of two genes (named *esterase A* and *esterase B*) or at least the *esterase B* [33–35]. Amplification of *esterase B1* in Californian *Culex* mosquitoes was the first event described in this context [36]. Variation in the number of copies among insects was also observed, being directly proportional to organophosphate resistance levels [37]. In agreement, laboratory insecticide selection pressure resulted in an increase in the gene copy numbers. However, it is likely that this process has a limit, since gene amplification is associated with a high fitness cost [38]. In fact, unequal crossing-over in the duplicated locus [37] may cause a reduction in copy number over time in the absence of insecticide pressure.

Gene duplication was also associated with another class of enzymes related to metabolic resistance, the multi-function oxidases (MFOs) or P450 [39]. Two genes of this class (*CYP6P9* and *CYP6P4*) were overexpressed in pyrethroid-resistant lineages of the malaria vector, *Anopheles funestus*. This overexpression is associated within *tandem* gene duplications, mapped in a quantitative trait locus (QTL locus *rp1*) and responsible for 87% of the genetic variation for pyrethroid resistance in this lineage. Besides, single nucleotide polimorphisms (SNPs) observed in these genes were described as insecticide-resistance markers [39]. Another gene duplication event was associated with overexpression of a P450 gene (*CYP9M10*) in a pyrethroid-resistant strain of *Culex quinquefasciatus* [40]. Duplications in genes coding for enzymes involved in metabolic resistance are somewhat expected, since they are components of supergene families bearing many paralogous genes, generally organized in genome clusters [41]. These are rapidly evolving families and few orthologs are identified among insect species [42]. In the *Ae. aegypti* genome, at least 26, 49 and 160 genes of the main detoxifying enzymes were identified corresponding, respectively, to GST, Esterases and MFO. These numbers represent an increase of 36% compared with *Anopheles gambiae* [43]. Recently, the importance of gene amplification for pyrethroid metabolic resistance was demonstrated in Caribbean *Ae. aegypti* populations. Compared with the susceptible strain, two genes (*CYP9J26* and the ABC transporter *ABCB4*) were amplified up to eight and seven copies, respectively [44].

Besides insecticide resistance, duplication of metabolic-resistance genes may also be selectively advantageous to the organism by increasing its general ability of detoxify xenobiotics. Moreover, new functions might be generated due to accumulation of substitutions in duplicated genes [45]. Such events would be more ‘free’ to occur, since the detoxifying enzyme system is redundant, reliant upon different enzymes with a similar function. Hence, the accumulation of potential loss of function alterations might not significantly compromise the metabolism [46].

By contrast, gene duplication events in molecules which are targets of neurotoxic insecticides are thought to be less likely, since they carry out very specific and essential activities, highly conserved throughout evolution. The increase in number might compromise the neurological functioning of the organism, an event described as dosage-balance hypothesis [47]. For instance, a *Culex pipiens* lineage with an acetilcolinesterase gene (*ace-1*) duplication presents 60% increase in enzyme activity. However, the acquired organophosphate resistance status is accompanied by an elevated cost of several life-history trait parameters [48]. Indeed, in a number of *Cx. pipiens* populations, the frequency of the *ace-1R*-mutant allele decays quickly in the absence of insecticide [49, 50], the same tendency observed for *ace-1R* in *An. gambiae* [51].

However, *Cx. pipiens*’ natural populations with a putative recent *ace-1* gene duplication (<40 years) have also been described. In these cases, both copies, with and without the mutation selected for organophosphate resistance, lie in the same chromosome. These mosquitoes, with a ‘heterozygous’ molecular phenotype, are resistant to organophosphates but have a lower fitness loss [52], suggesting a mechanism which favors the occurrence of duplications in neurotoxic insecticide target-coding genes.

Herein, we initially hypothesized a duplication in a region of the *Na_V_* gene of *Ae. aegypti* (*AaNa_V_*) as a polymorphic trait in natural populations of this important vector, which would include one-mutant haplotype for the 1011 site together with one wild-type for both sites, 1011Met + 1016Val and 1011Ile + 1016Val, respectively, supported by a fund of evidence. AS-PCR genotyping confirmed that all individuals carrying the 1011Met mutation were (phenotypically) ‘heterozygous’. In addition, sequencing of the *AaNa_V_* IIS6 genomic region revealed some individuals with three haplotypes, suggesting the existence of a duplication with the proposed aforementioned composition. Similar results of mosquitoes harboring three alleles were recently reported for the *An. gambiae* acetilcolinesterase *ace-1* gene and interpreted as evidence of a gene duplication event [53].

Saavedra-Rodriguez *et al.* [16] evaluated the role of *AaNa_V_* mutations in pyrethroid resistance by analyzing the susceptibility of the F3 offspring from the parental crossing ♀1011Ile/Met + 1016Ile/Ile (from Isla Mujeres, Mexico) × ♂1011Ile/Ile + 1016Val/Val (from New Orleans, lineage control of susceptibility). Interestingly, if the presence of a duplicated sodium channel had been considered, interpretation of some results would have been made easier since they would have better explained the different genotypes in the crosses. In addition, it is remarkable that the Ile1011Met substitution seems to appear in ‘homozygosis’ (1011Met/Met) in high frequency in other localities in Latin America [16, 54], indicating that this mutation is not recessive-lethal and that different types of duplicated haplotypes probably coexist in *Ae. aegypti* populations. This might also suggest that the gene duplication in the *Ae. aegypti Na_V_* gene we observed in Brazilian populations is a relatively recent event.

Our initial hypothesis was that, at least for the *Ae. aegypti* populations studied herein, the 1011Met mutation occurs only in a duplicated haplotype containing a type ‘A’ sequence and the 1016Val wild-type allele, together and in linkage disequilibrium with a type ‘B’ sequence, containing the wild-type allele for both the 1011 and 1016 positions (Fig. 2). The high frequency of ‘heterozygous’ A/B, the lack of 1011Met/Met specimens, 1011Ile/Met + 1016Ile/Ile genotypes and the molecular phenotype of the offspring analyzed here support this hypothesis. However, the results obtained by the copy number variation assay show a ratio of five copies of the *AaNa_V_* gene in the EE-selected lineage when compared with the Rockefeller strain, indicating that further duplication events might have taken place, possibly as a result of unequal crossing-over. Moreover, it is presumed that the number of copies is a polymorphic trait, given the large variation observed when using single mosquito DNA (data not shown), which was diminished when pools of 10 larvae were employed. The variation in the number of copies in natural populations remains to be investigated as an important clue for this evolutionary process.

Amplification of the *Na_V_* gene was also recently demonstrated in a pyrethroid*-*resistant *C. quinquefasciatus* lineage. The classical *kdr* mutation (Leu1014Phe), strongly associated to pyrethroid resistance, was present in one type of sequence. The other type of sequence lacked the intron close to the 1014 site and was not related to resistance. This haplotype was suggested to be a pseudogene [55].

To the best of our knowledge, we present here the first evidence of a duplication event in the sodium channel gene of the dengue vector, *Ae. aegypti*. Although the available data point to a more important role of the mutations in the 1016 site for pyrethroid resistance, there is clear evidence that the 1011Met mutation, which is associated with the duplication/amplification event(s), is also associated with some resistance [12, 14]. Therefore, the gene duplication and amplification in the *Ae. aegypti Na_V_* gene might be a recent adaptive response to the intense use of insecticides, maintaining together wild-type and mutant alleles in the same organism conferring some resistance at the same time as reducing some of its deleterious effects on other aspects of fitness. It will be very interesting to investigate how much diversity in copy number variation there is in natural populations, besides its possible association with pyrethroid resistance and fitness cost. It is also intriguing whether the mosquito sodium channel gene is more prone to duplications than that of other pyrethroid-selected insects as well as what the potential evolutionary interpretation and implications of this process are.

## SUPPLEMENTARY DATA

Supplementary data is available at *EMPH* online.

Supplementary Data
