# Supplementary material for: Evidence for gene duplication in the voltage-gated sodium channel gene of Aedes aegypti
Source: Evol Med Public Health. 2013 Jun 19;2013(1):148–60. doi: 10.1093/emph/eot012 (PMC3868448; doi:10.1093/emph/eot012)
Supplement: Supplementary Data [file supp_2013_1_148__index.html]

Evidence for gene duplication in the voltage-gated sodium channel gene of Aedes aegypti — Supplementary Data 

# Evidence for gene duplication in the voltage-gated sodium channel gene of *Aedes aegypti*

## 

files

**Files in this Data Supplement:**

- Supplementary Data - pdf file
