# Supplementary material for: Evidence for gene duplication in the voltage-gated sodium channel gene of Aedes aegypti
Source: Evol Med Public Health. 2013 Jun 19;2013(1):148–60. doi: 10.1093/emph/eot012 (PMC3868448; doi:10.1093/emph/eot012)
Supplement: Supplementary Data [file supp_eot012_Table_S1.pdf]

Table S1 – Raw data for real time TaqMan copy number assays.

Assays	samples	AaNaV IIS6			RP49			ΔCт (Mean)	μΔCт(Mean)	ΔΔCт
		Cт (Mean)	Cт SD	Ct Threshold	Cт (Mean)	Cт SD	Ct Threshold			
assay #1	Rock-1	21,84	0,03	0,101	24,57	0,03	0,202	-0,37		
	Rock-2	22,12	0,22		24,81	0,16		-0,50	-0,40	0,00
	Rock-3	21,82	0,12		24,50	0,12		-0,33		
	EE-1	24,12	0,28		24,49	0,20		-2,73		
	EE-2	23,86	0,08		24,36	0,05		-2,69	-2,70	-2,30
	EE-3	23,91	0,09		24,24	0,04		-2,68		
	RockxEE(F1)-1	22,62	0,08		24,49	0,06		-1,87		
	RockxEE(F1)-2	22,64	0,05		24,64	0,03		-2,00	-1,96	-1,56
	RockxEE(F1)-3	22,75	0,03		24,75	0,05		-1,99		
assay #2	Rock-1	24,05	0,10	0,105	24,04	0,02	0,177	0,00		
	Rock-2	23,95	0,03		24,13	0,04		-0,18	-0,05	0,00
	Rock-3	23,99	0,11		23,96	0,06		0,03		
	EE-1	21,91	0,04		24,31	0,05		-2,40		
	EE-2	21,60	0,09		23,98	0,03		-2,38	-2,41	-2,37
	EE-3	21,83	0,06		24,28	0,06		-2,46		
	RockxEE(F1)-1	22,65	0,09		24,29	0,06		-1,65		
	RockxEE(F1)-2	22,77	0,11		24,52	0,12		-1,74	-1,68	-1,63
	RockxEE(F1)-3	22,72	0,01		24,37	0,05		-1,66		
assay #3	Rock-1	23,89	0,04	0,095	24,60	0,04	0,222	-0,71		
	Rock-2	23,97	0,15		24,72	0,08		-0,76	-0,69	0,00
	Rock-3	23,90	0,04		24,52	0,05		-0,61		
	EE-1	21,76	0,05		24,85	0,05		-3,09		
	EE-2	21,46	0,05		24,47	0,07		-3,02	-3,05	-2,36
	EE-3	21,73	0,07		24,77	0,05		-3,04		
	RockxEE(F1)-1	22,56	0,04		24,88	0,06		-2,33		
	RockxEE(F1)-2	22,44	0,07		24,88	0,09		-2,44	-2,40	-1,70
	RockxEE(F1)-3	22,52	0,06		24,94	0,03		-2,42		

Each sample consisted of DNA extracted from a ten larvae pool. Values for Cт (Means) and Cт (SD) correspond to the mean and standard deviation of three replicas of each sample, for both $AaNa_V$ IIS6 region and RP49 targets, respectively. Indicated Cт tresholds were automatically set up by the software for each assay. The ΔΔCт values were used to calculate the copy number variation (see Table 3).
